# A cross-sectional and longitudinal cohort study of creatinine-to-cystatin C ratio and cardiovascular disease risk in a middle-aged and elderly population

**DOI:** 10.3389/fendo.2025.1531394

**Published:** 2025-05-23

**Authors:** Yuling Chen, Fengmin Xu, Jia Li, Yixi Bao

**Affiliations:** ^1^ Department of Clinical Laboratory, The Second Affiliated Hospital of Chongqing Medical University, Chongqing, China; ^2^ Department of Clinical Laboratory, Beijing Anzhen Nanchong Hospital of Capital Medical University & Nanchong Central Hospital, The Second Clinical Medical College of North Sichuan Medical College, Nanchong, Sichuan, China

**Keywords:** creatinine, cystatin C, cardiovascular disease, older adults, CCR

## Abstract

**Background:**

The creatinine-to-cystatin C ratio (CCR) has recently been proposed as a proxy indicator for sarcopenia. It has been linked to a range of adverse outcomes. However, the relationship between the CCR and cardiovascular disease (CVD) is not widely recognized. This study used data from the China Health and Retirement Longitudinal Study to investigate the association between the CCR and CVD in a middle-aged and elderly population.

**Methods:**

The cross-sectional study and longitudinal cohort study included 10,614 and 6,720 passengers, respectively. The occurrence of CVD incidents was defined as self-reported health history or receipt of cardiac disease treatment. The CCR through creatinine (mg/dL) and cystatin C (mg/dL) were calculated and grouped by quartiles. Unadjusted and adjusted logistic regression models were employed to further explore the CCR-CVD relationships.

**Results:**

The findings of our study demonstrated a progressively significant reduction in the risk of CVD with an additional CCR. The cross-sectional cohort findings indicated a 21% reduction in the risk of CVD with every additional unit of CCR (OR=0.79, 95% CI, 0.73-0.84). In three logistic regression models, there was a significant association between CCR quartiles and a lower risk of CCR (p for trend <0.001). Further subgroup analyses revealed a 16% reduction in the incidence of CVD with each additional unit of CCR among individuals aged below 65 years (OR, 0.84; 95% CI, 0.78–0.91) and a 18% decline in CVD with each unit of CCR in married populations (OR, 0.82; 95% CI, 0.77–0.88). The findings of the Longitudinal Cohort Study indicated that for each unit increase in CCR, there was an 22% reduction in the risk of CVD (OR=0.78, 95% CI, 0.68-0.90). In logistic regression models adjusted for all co-dependent variables, the prevalence of CVD was reduced by 15%, 21%, and 41% as the number of CCR quartiles increased. This result was also verified by restricted cubic spline analysis.

**Conclusion:**

In conclusion, the correlation between an elevated CCR and a reduced risk of CVD in middle-aged and elderly populations has been established. Enhanced CCR levels may prove useful in predicting CVD occurrence in the elderly, thus representing a simple and effective biomarker.

## 1 Introduction

Cardiovascular disease (CVD) represents a significant global health burden, contributing to a considerable proportion of deaths (1). Statistical data indicates that CVD is the underlying cause of one-third of all global deaths (2). Despite the long-standing focus on conventional CVD risk factors (e.g. hypertension, diabetes mellitus, high cholesterol, etc.), the incidence of both CVDs and cardiovascular deaths has continued to increase as the population continues to age, particularly in this elderly population group (3). In order to most effectively address this complex situation, investigators have progressively examined a number of previously overlooked emerging risk factors, with particular attention being paid to sarcopenia, specifically in relation to the understanding of muscle loss and frailty in patients with CVDs (4).

Sarcopenia is an age-related condition characterized by progressive and systemic musculoskeletal disorders, resulting in a loss of muscle quality and tone. It is a common occurrence in the elderly population and is associated with an increased risk of adverse outcomes (5). In addition to its effects on physical functioning and quality of life, sarcopenia is also associated with a range of meteorological abnormalities, including oxidative stress and chronic inflammation (6–8). Such anomalies are strongly linked to the onset, evolution and prognosis of a variety of chronic diseases (9, 10), particularly in light of the growing body of evidence examining their relevance to CVD health. In recent years, a substantial and continually increasing body of evidence has investigated the correlation between sarcopenia and conditions associated with CVD, including coronary heart disease, atrial fibrillation, heart failure, and stroke (11–14).

The diagnosis of sarcopenia necessitates a comprehensive evaluation encompassing muscle mass, strength, and physical performance. Conventionally employed metrics such as DXA, CT, BIA, and MRI, despite their efficacy in quantifying muscle mass, are encumbered by inherent limitations including the expense of equipment, the intricacy of operation, the challenge of dynamic monitoring, and the inability to facilitate early detection. While these metrics offer precise measurements of muscle mass, their application is hindered by factors such as the cost of equipment, the complexity of operation, the difficulty in dynamic monitoring, and the challenge in early detection (15, 16). There is an urgent requirement for the development of simple and cost-effective biomarkers for the diagnosis and surveillance of muscle loss disorders. In recent times, the creatinine-to-cystatin C ratio (CCR) has been put forth as a dependable surrogate for the evaluation of secondary muscle mass, given its accessibility and cost-effectiveness as a biomarker (17, 18). CCR is regarded as a promising predictor of sarcopenia, offering a number of advantages over traditional methods in terms of feasibility and affordability. Furthermore, studies have demonstrated a clear relationship between CCR and muscle mass (19, 20).

Despite the existence of research investigating the associations between sarcopenia and CVD, the majority of studies are based largely on cross-sectional analyses. Furthermore, there is considerable variation in the criteria used to determine sarcopenia, and the role of sarcopenia as an independent risk factor for CVD remains uncertain (12, 14, 21). With the gradual introduction of CCR as a biomarker for evaluating muscle mass and fiber, its potential in CVD disease risk evaluation ought to be investigated much further.

A plethora of prior studies have validated the standalone correlation of creatinine (Cre) and cystatin C (Cysc) with CVD. Nevertheless, there persists a dearth of substantial knowledge concerning the research on the ratio (CCR) of these two proteins. Firstly, extant literature has focused primarily on the analysis of individual markers (22, 23), neglecting to thoroughly explore the distinctive clinical significance that the CCR, as an integrative indicator, might offer. Secondly, there is a paucity of cross-sectional and longitudinal cohort studies that specifically emphasize the relationship between the CCR and CVD. The existing studies have led to an inadequate body of evidence to support causal inferences regarding its predictive value. It is imperative to note that, despite the theoretical possibility of a relationship between muscle metabolic status and CCR levels, no studies have been conducted to systematically assess the independent predictive role of CCR on CVD risk. This assessment would be based on controlling for conventional cardiovascular risk factors, including BMI, glucose, lipids, and renal impairment. The existence of these critical issues necessitates further validation of the value of CCR as a potential biomarker in clinical practice.

Consequently, the present study employs a combination of cross-sectional and longitudinal cohort studies, with full adjustment for confounders such as body mass index (BMI), triglycerides (TG), total cholesterol (TC), blood glucose (Glu), estimated glomerular filtration rate (eGFR), among others. This approach aims to comprehensively investigate the relationship between CCR and CVD. The objective is to validate the potential of CCR as an emerging biomarker for CVD prediction.

## 2 Materials and methods

### 2.1 Data sources

CHARLS (China Health and Retirement Longitudinal Study) is a nation-wide, longitudinal, forward-looking survey that aims to research the socioeconomic and physical health conditions of communities of Chinese people aged 45 and over, as well as the influential factors on these outcomes (24). The survey was initiated by the National Development Research Institute of Peking University and funded by the U.S. National Institute on Aging, with data collection commencing in 2011 (24). The initial baseline investigation, completed in 2011, involved 17,708 participants in 150 counties and 450 communities across 28 provinces in China (24). The survey was comprehensive in scope, encompassing a wide range of topics, including population demographics, family composition, health profile, physical functionality, healthcare utilization, revenue, and expenses. Subsequently, the CHARLS has conducted follow-up visits every two to three years, resulting in the publication of five survey waves: in 2011, 2013, 2015, 2018, and 2020. The follow-up surveys have been subject to rigorous quality control procedures, with all appointments conducted by trainee surveyors utilizing a computer-assisted personal interviewing system. In order to guarantee the ethical conformity of the investigation, the process of data collection and the conduct of the study in CHARLS was approved by the Ethics Committee of Peking University (IRB 00001052-11015), and written informed consent was obtained from all participants (24).

### 2.2 Study population

**Figure 1:**
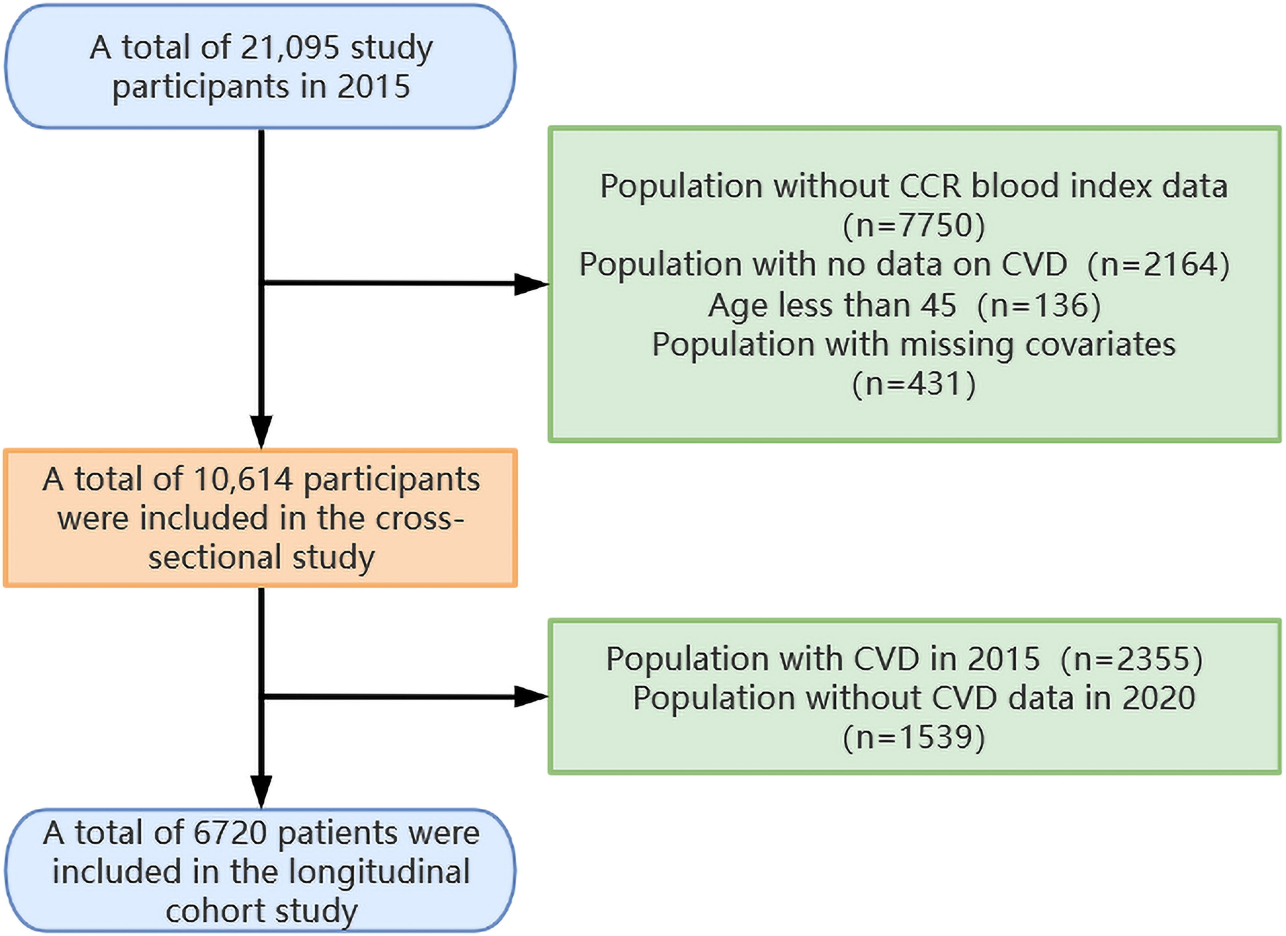
Experimental procedure for this study.

The research procedure has been outlined in **Figure 1**. The current blood examination data were derived from two distinct data sets: Wave 1 (baseline time, 2011) and Wave 3 (follow-up time, 2015). Given the larger number of blood sampling results available for Wave 3, this data set was selected as the baseline for the current analysis. A total of 21,095 subjects were enrolled in the research in 2015. Patients who had no data on CCR blood markers (n=7750), no CVD data (n=2164), age <45 years (n=136), and covariate missing (n=431) were excluded from the cross-sectional study. The remaining patients, comprising 10,614 individuals, were entered into the cross-sectional study. Of these, patients with no CVD in 2015 (n=8259) were incorporated into the longitudinal cohort study. The longitudinal cohort study employed 2015 as the baseline data and 2020 as the end point data. It included qualified patients who did not have CVD in 2015 (n=8,259) and excluded patients who had no CVD data in 2020 (n=1,539). This resulted in the enrollment of 6,720 patients in the longitudinal cohort study.

### 2.3 Diagnostic criteria for CVD

The term “self-reported history of CVD” is defined as a history of CVD that is reported by the individual in question. (i) either self-reported in response to the question, ‘Have you been diagnosed by a doctor as having a stroke/heart attack?’; (ii) deducible in response to the question, ‘Are you currently receiving any of the following treatments (Traditional Chinese Medicine/Western Medicine/Other treatments/None of the above) for stroke/heart attack or for its difficulties?’. The aforementioned criteria were then applied, resulting in the categorization of participants as patients with CVD. This included those who had been diagnosed with heart disease or stroke, as well as those who had undergone specialized treatment for these conditions.

### 2.4 Dependent variable CCR

A team of specially trained staff members collected blood specimens from all interviewees. Plasma was separated and transported to Beijing, where it was stored at -20°C. The samples were then analyzed at the Chinese Center for Disease Control and Prevention (24). The staff used standard methods to analyze participants’ intravenous plasma samples for TG, TC, high-density lipoprotein cholesterol (HDL-C), Glu, Cysc, and Cre.

The estimated CCR was calculated by using the formula below:


$$CCR=\frac{\text{Cre}\left(mg/dL\right)}{\text{Cysc}\left(mg/dL\right)}$$

### 2.5 Covariates

Potentially confounding factors that may be associated with CVD were identified as covariates, including demographic variables (age, gender, educational level, locality and marital status), anthropometric variables (TG, TC, Glu, eGFR, BMI, systolic blood pressure (SBP) and diastolic blood pressure (DBP), with the latter being calculated as the mean of three blood pressure measurements) and health-related behavioral variables (consumption habits of alcohol and smoking status).

The estimated eGFR was calculated by using the formula below (25, 26):


$$\text{Male}:\text{eGFR}=141\;*\text{ min }{\left(\text{Cre}/0.9,\;1\right)}^{-\;0.411}*\text{ max }{\left(\text{Cre}/0.9,\;1\right)}^{-\;1.209}*\;0.993^{\text{Age}}$$

$$\text{Female}:\text{eGFR}=141\;*\text{ min }{\left(\text{Cre}/0.7,\;1\right)}^{-\;0.329}*\text{ max }{\left(\text{Cre}/0.7,\;1\right)}^{-\;1.209}*\;0.993^{\text{Age}}*\;1.018$$

### 2.6 Statistical analysis

The data from this study were subjected to analysis using a number of different statistical methods. In the case of quantitative variables that did not follow a normal distribution, medians and interquartile ranges (IQR) were employed to characterize them. Conversely, for variables that were normally distributed, means and standard deviations (SD) were utilized. Qualitative variables were presented by counts and percentages. The significance of between-group differences was evaluated using the Wilcoxon rank sum test, one-way ANOVA, or Pearson chi-square test. To ensure the absence of notable covariances among covariates, multicollinearity was assessed through the calculation of tolerance and variance inflation factor (VIF). All VIFs were found to be less than 5, indicating the absence of multicollinearity (27). The participants were grouped into quartiles of the CCR (Q1 to Q4) for the purpose of comparing the baseline characteristics and cardiovascular morbidity. Three univariate and multivariate logistic regression models were constructed. The model 1 was an unmodified single-variable logistic regression model; model 2 was an adjusted model for age, gender, educational level, locality, and marital status; and model 3 was a further adjusted model based on model 2 with the additional variables of BMI, smoking status, alcohol consumption, blood pressure, TG, TC, Glu and eGFR. The results were presented as odds ratios (ORs) with 95% confidence intervals (CIs). Furthermore, a multivariate-adjusted restricted cubic spline (RCS) analysis was employed to assess the dose-response relationship between CCR and CVD. Finally, subgroup and interactional analyses were conducted to explore the associations in various subgroups, including factors such as age, gender, education level, location, marital status, smoking, and alcohol consumption. All statistical analyses were conducted using the R software package, and a two-sided p-value of less than 0.05 was considered statistically significant.

## 3 Results

### 3.1 Results of the 2015 cross-sectional study

#### 3.1.1 Baseline characteristics of participants

The characteristics of the research population were presented in **Table 1**. The total number of participants was 10,614, with a median age of 61.35 years. Of these, 5,660 (53.3%) were female and 4,954 (46.7%) were male. Among the participants, 2,355 (22.2%) had a history of CVD. The baseline median (IQR) CCR for all participants was 9.56 (6.92, 12.20). Compared to those without CVD, participants with CVD exhibited significantly elevated levels of SBP, DBP, BMI, TG, GLU, Cre, and Cysc (p < 0.05). Conversely, significantly reduced CCR, HDL-C and eGFR were observed in those subjects who suffered from CVD (p < 0.001).

**Table 1:** Baseline characteristics of Cross-Sectional study participants.

Variable	Total (n = 10, 614)	Non-CVD (n = 8,259)	CVD (n = 2,355)	*P*
Age	61.35 (9.19)	60.71 (9.14)	63.58 (9.02)	<0.001
Gender				
Female	5660 (53.3)	4296 (52.0)	1364 (57.9)	<0.001
Male	4954 (46.7)	3963 (48.0)	991 (42.1)	
Education				
College or above	380 (3.6)	261 (3.2)	119 (5.1)	<0.001
Primary school or below	7226 (68.1)	5611 (67.9)	1615 (68.6)	
Secondary school	3008 (28.3)	2387 (28.9)	621 (26.4)	
Marital				
Married	9148 (86.2)	7187 (87.0)	1961 (83.3)	<0.001
Non-married	1466 (13.8)	1072 (13.0)	394 (16.7)	
Location				
City/town	3850 (36.3)	2895 (35.1)	955 (40.6)	<0.001
Village	6764 (63.7)	5364 (64.9)	1400 (59.4)	
Smoke				
Current smoker	2940 (27.7)	2419 (29.3)	521 (22.1)	<0.001
Ex-smoker	1721 (16.2)	1230 (14.9)	491 (20.8)	
Non-smoker	5953 (56.1)	4610 (55.8)	1343 (57.0)	
Drink				
Drink but less than once a month	922 (8.7)	727 (8.8)	195 (8.3)	<0.001
Drink more than once a month	2733 (25.7)	2274 (27.5)	459 (19.5)	
None of these	6959 (65.6)	5258 (63.7)	1701 (72.2)	
SBP (mmHg)	128.93 (20.04)	127.75 (19.71)	133.10 (20.62)	<0.001
DBP (mmHg)	75.64 (11.71)	75.32 (11.63)	76.79 (11.95)	<0.001
BMI (kg/m2)	23.93 (4.07)	23.67 (3.86)	24.81 (4.63)	<0.001
TG (mg/dL)	142.67 (90.49)	139.93 (89.32)	152.28 (93.88)	<0.001
TC (mg/dL)	184.33 (36.23)	184.08 (36.08)	185.23 (36.77)	0.171
HDL (mg/dL)	51.29 (11.60)	51.65 (11.58)	50.02 (11.60)	<0.001
GLU (mg/dL)	103.98 (36.09)	103.45 (35.33)	105.84 (38.59)	0.005
eGFR (mL/min)	89.20 (16.25)	90.02 (15.98)	86.32 (16.88)	<0.001
Cre (mg/dL)	0.81 (0.30)	0.81 (0.30)	0.82 (0.30)	0.033
Cysc (mg/dL)	0.09 (0.02)	0.08 (0.02)	0.09 (0.03)	<0.001
CCR	9.56 (2.64)	9.69 (2.72)	9.12 (2.28)	<0.001
Quartiles of CCR Q1	2654 (25.0)	1895 (22.9)	759 (32.2)	<0.001
Q2	2653 (25.0)	2070 (25.1)	583 (24.8)	
Q3	2653 (25.0)	2082 (25.2)	571 (24.2)	
Q4	2654 (25.0)	2212 (26.8)	442 (18.8)	

#### 3.1.2 Relationship between CCR and the incidence of CVDs

Prior to establishing regression models, multicollinearity among the variables was evaluated, with the results indicating that the VIFs were all below 5 (see **Supplementary Table 1**). This suggested that there was no significant covariation among the variables. The correlations between CCR quartile groupings and continuum variables with the incidence of CVD were detailed in **Table 2**. A marked reduction in the prevalence of CVD was observed in the quartile subgroups (p for trend <0.001). The lowest risk of CVD was observed in the fourth CCR quartile (Q4) across all three models (p for trend <0.001). In both models 2 and 3, which were adjusted for covariates, the risk of disease was reduced in Q2 in comparison with Q3 (p for trend <0.001). There was a tendency for a significant inverse correlation between CCR and the occurrence of CVD when it was considered a covariate. For each IQR increase in CCR, the risk of CVD decreased by 21% (OR=0.79, 95% CI, 0.73-0.84), with a p-value of less than 0.001.

**Table 2:** Odds ratio between CCR and CVD risk.

CCR and CVD	Model 1	*P*	Model 2	*P*	Model 3	*P*
CCR per IQR	0.77 [0.81,0.92]	<0.001	0.85 [0.80,0.90]	<0.001	0.79 [0.73,0.84]	<0.001
Quartiles of CCR						
Q1	Reference		Reference		Reference	
Q2	0.70 [0.62,0.80]	<0.001	0.79 [0.69,0.90]	<0.001	0.79 [0.69,0.91]	<0.001
Q3	0.68 [0.60,0.78]	<0.001	0.81 [0.71,0.93]	0.003	0.80 [0.69,0.93]	0.003
Q4	0.50 [0.44,0.57]	<0.001	0.64 [0.55,0.75]	<0.001	0.60 [0.51,0.71]	<0.001
p for trend	<0.001		<0.001		<0.001	

**Figure 2:**
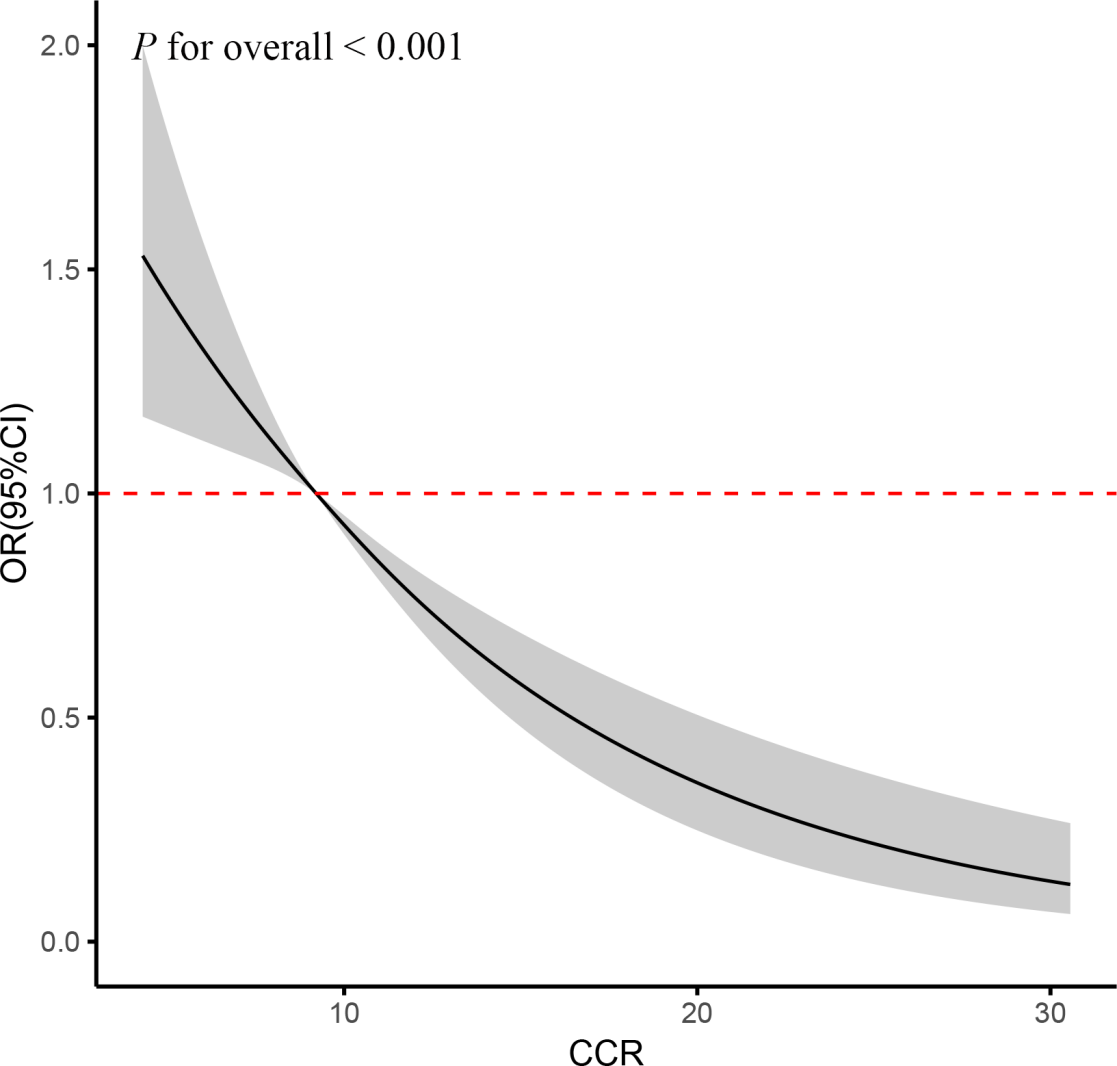
RCS curve of the association between CCR and CVD risk. It was adjusted for age, gender, education, location and marital status, body mass index, smoking status, drinking status, blood pressure, TG, TC, Glu, and eGFR. P for overall <0.001: Nonlinear significance between CCR and CVD.

The fully adjusted RCS regression analyses revealed a statistically significant linear relationship between accumulated CCR and CVD incidence (P for overall <0.001) (**Figure 2**). The results indicated a negative linear coefficient of interest between CCR and CVD risk.

#### 3.1.3 Subgroup analysis

**Figure 3:**
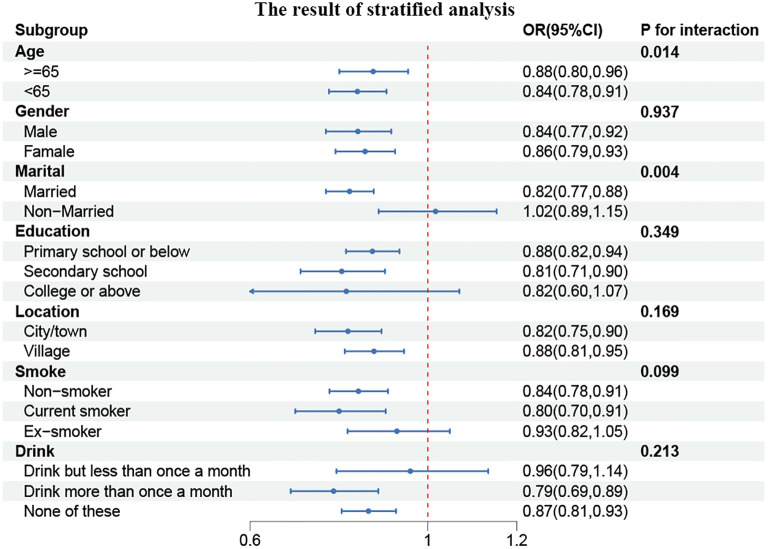
Forest plot of stratified analyses of the correlation between CCR and CVD risk. P for interaction <0.05: Statistical difference between subgroups.

To gain further insight into the relationship between CCR and CVD prevalence, we conducted subgroup analyses of the parameters among participants stratified according to their characteristics. As illustrated in **Figure 3**, a statistically significant correlation was observed between CCR and age and marital status (P for interaction <0.05). In individuals under the age of 65, the prevalence of CVD exhibited a 16% reduction for each additional unit of CCR (OR, 0.84; 95% CI, 0.78–0.91). Among the married population, the prevalence of CVD demonstrated a 18% reduction for each unit of CCR (OR, 0.82; 95% CI, 0.77–0.88).

### 3.2 Results of the 2015–2020 longitudinal cohort study

#### 3.2.1 Baseline characteristics of participants

The characteristics of the population under study were presented in **Table 3**. A total of 6720 participants were enrolled in the study, with a median age of 60.03 years, 3557 (52.9%) of whom were female and 3163 (47.1%) male. Of these participants, 541 (8.05%) subsequently developed CVD. The median (IQR) CCR at baseline was 9.75 (7.01, 12.49) for all participants. The trends observed in the baseline outcomes were consistent with those seen in the cross-sectional results. Subjects who developed CVD exhibited considerably elevated levels of SBP, DBP, BMI, TC, GLU, and Cysc (p < 0.05) and a markedly reduced CCR (p < 0.001) in comparison to those who did not develop CVD.

**Table 3:** Baseline characteristics of longitudinal cohort studies participants.

Variable	Total (n = 6, 720)	Non-CVD (n = 6,179)	CVD (n = 541)	*P*
Age	60.03 (8.86)	59.80 (8.85)	62.61 (8.49)	<0.001
Gender				
Female	3557 (52.9)	3262 (52.8)	295 (54.5)	0.465
Male	3163 (47.1)	2917 (47.2)	246 (45.5)	
Education				
College or above	205 (3.1)	191 (3.1)	14 (2.6)	0.014
Primary school or below	4541 (67.6)	4145 (67.1)	396 (73.2)	
Secondary school	1974 (29.4)	1843 (29.8)	131 (24.2)	
Marital				
Married	5927 (88.2)	5464 (88.4)	463 (85.6)	0.058
Non-married	793 (11.8)	715 (11.6)	78 (14.4)	
Location				
City/town	2282 (34.0)	2102 (34.0)	180 (33.3)	0.761
Village	4438 (66.0)	4077 (66.0)	361 (66.7)	
Smoke				
Current smoker	1942 (28.9)	1800 (29.1)	142 (26.2)	0.136
Ex-smoker	940 (14.0)	851 (13.8)	89 (16.5)	
Non-smoker	3838 (57.1)	3528 (57.1)	310 (57.3)	
Drink				
Drink but less than once a month	598 (8.9)	551 (8.9)	47 (8.7)	0.149
Drink more than once a month	1872 (27.9)	1740 (28.2)	132 (24.4)	
None of these	4250 (63.2)	3888 (62.9)	362 (66.9)	
SBP (mmHg)	126.79 (19.20)	126.27 (19.01)	132.67 (20.29)	<0.001
DBP (mmHg)	75.08 (11.59)	74.88 (11.45)	77.29 (12.83)	<0.001
BMI (kg/m2)	23.67 (3.72)	23.64 (3.71)	24.01 (3.86)	0.027
TG (mg/dL)	139.58 (88.70)	139.10 (88.77)	145.07 (87.79)	0.133
TC (mg/dL)	184.09 (36.08)	183.73 (36.14)	188.15 (35.21)	0.006
HDL (mg/dL)	51.82 (11.47)	51.79 (11.47)	52.18 (11.44)	0.452
GLU (mg/dL)	102.56 (33.18)	102.15 (32.46)	107.30 (40.21)	0.001
eGFR (mL/min)	90.70 (15.57)	90.84 (15.59)	89.15 (15.25)	0.015
Cre (mg/dL)	0.80 (0.26)	0.80 (0.26)	0.80 (0.26)	0.824
Cysc (mg/dL)	0.08 (0.02)	0.08 (0.02)	0.09 (0.02)	<0.001
CCR	9.75 (2.74)	9.80 (2.77)	9.19 (2.27)	<0.001
Quartiles of CCR Q1	1680 (25.0)	1501 (24.3)	179 (33.1)	<0.001
Q2	1680 (25.0)	1541 (24.9)	139 (25.7)	
Q3	1680 (25.0)	1553 (25.1)	127 (23.5)	
Q4	1680 (25.0)	1584 (25.6)	96 (17.7)	

#### 3.2.2 Relationship between CCR and the incidence of CVDs

Similarly, the presence of multicollinearity among the variables was evaluated, and the results demonstrated that the VIFs were all below 5 (see **Supplementary Table 1**). This finding suggested that no substantial multicollinearity exists among the variables. **Table 4** provided a detailed elaboration of the CCR’s interquartile groupings and correlations between continuous variables and the incidence of CVD. The longitudinal cohort resulted exhibited a consistent trend with those of the cross-sectional analysis. A statistically significant trend of reduced CVD incidence was observed in both the continuous variable and quartile groupings when the CCR was considered as a continuous variable (p for trend <0.001). For each additional IQR of CCR, the risk of CVD was reduced by 22%(OR = 0.78, 95% CI = 0.68–0.90) (p < 0.001). The lowest risk of CVD was observed in the fourth quartile of the three models (p for trend < 0.001). In Model 3, which was adjusted for all covariates, the prevalence of CVD exhibited a decline with increasing CCR quartiles, with an OR (95% CI) of 0.85 (0.66, 1.09), 0.79 (0.60, 1.03), and 0.59 (0.42, 0.83), respectively (p for trend = 0.002). In other words, as the number of CCR quartiles increased, the incidence of CVD was reduced by 15%, 21%, and 41%, respectively.

**Table 4:** Odds ratio between CCR and CVD risk.

CCR and CVD	Model 1	*P*	Model 2	*P*	Model 3	*P*
CCR per IQR	0.75 [0.67,0.84]	<0.001	0.80 [0.71,0.90]	<0.001	0.78 [0.68,0.90]	<0.001
Quartiles of CCR						
Q1	Reference		Reference		Reference	
Q2	0.76 [0.60,0.95]	0.019	0.81 [0.64,1.03]	0.083	0.85 [0.66,1.09]	0.193
Q3	0.69 [0.54,0.87]	0.002	0.75 [0.58,0.96]	0.026	0.79 [0.60,1.03]	0.084
Q4	0.51 [0.39,0.66]	<0.001	0.58 [0.43,0.77]	<0.001	0.59 [0.42,0.83]	0.002
p for trend	<0.001		<0.001		0.002	

**Figure 4:**
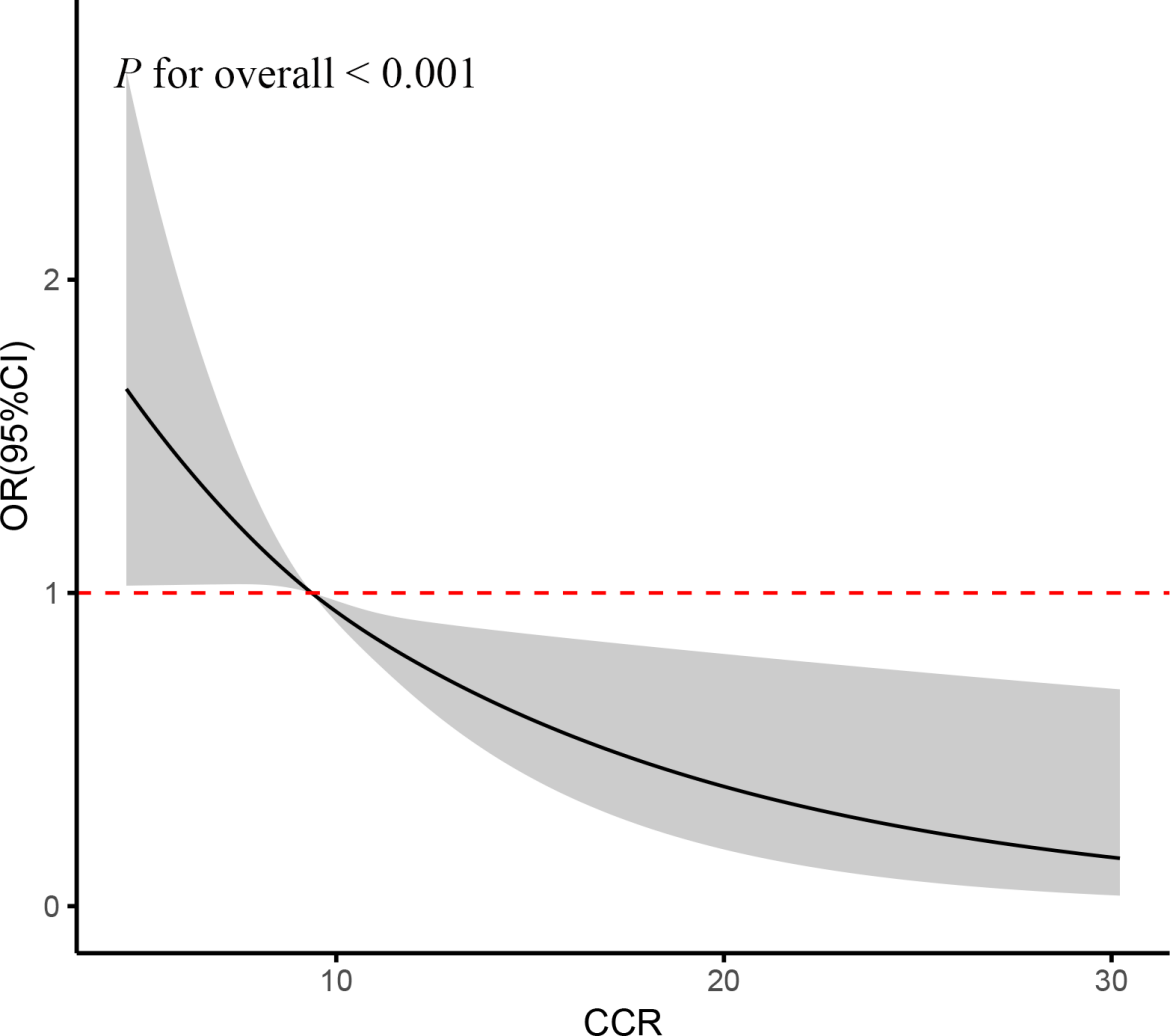
RCS curve of the association between CCR and CVD risk. It was adjusted for age, gender, education, location and marital status, body mass index, smoking status, drinking status, blood pressure, TG, TC, Glu, and eGFR.

The fully adjusted RCS regression models indicated a negative linear association between cumulative CCR and CVD incidence (P for overall < 0.001) (**Figure 4**). In alignment with the cross-sectional findings, this signified a linear inverse correlation between CCR and CVD risk.

#### 3.2.3 Subgroup analysis

**Figure 5:**
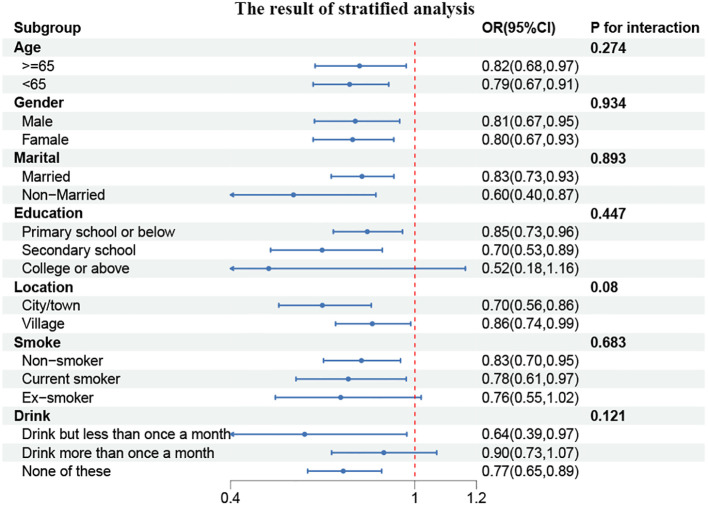
Forest plot of stratified analyses of the correlation between CCR and CVD risk.

Similarly, the participants were divided into subgroups based on their characteristics, and the resulting data were analyzed. It was found that none of the subgroups, including age, gender, marital status, education, place of residence, smoking status, or alcohol consumption, significantly altered the relationship between CCR and the incidence of cardiovascular disease, as illustrated in **Figure 5** (P for interaction > 0.05). However, a consistent effect trend was observed in comparison to the cross-sectional data.

## 4 Discussion

The results of this comprehensive study demonstrated a consistency between cross-sectional and longitudinal cohort studies. Baseline levels of CCR were observed to be lower in patients with CVD (p < 0.001). This study was the first to demonstrate that CCR continues to exhibit an independent association with cardiovascular risk, even after adjusted for conventional cardiovascular risk factors, particularly in those over 65 years of age. In other words, the risk of CVD increased as the level of CCR decreased, and vice versa, the risk of CVD was lower in those with higher levels of CCR. Restrictive triple spline analysis also validated this result. The present study confirmed for the first time the validity of CCR as a biomarker for assessing the prevalence of CVD. This finding has significant implications for the assessment of cardiovascular risk in the elderly population. Despite its inability to fully supplant imaging, CCR may serve as a viable primary screening instrument or a dynamic monitoring indicator, offering a novel approach to cardiovascular health management.

A growing body of evidence from numerous studies has demonstrated a correlation between sarcopenia and CVD, particularly in the elderly population (28, 29). Furthermore, sarcopenia has been identified as a risk factor for the progression of CVD (30). A population-based study of middle-aged and older Chinese adults with sarcopenia, defined as low levels of strength, mass and fitness, identified a strong link with an increased risk of CVD, including heart disease and stroke (21). However, the majority of studies have concentrated on either muscle mass or a single factor of muscular function, with no suitable metrics available for clinical assessment. In comparison, we introduced the CCR as a complex indicator that assesses the impact of muscular health on CVD risk in a more holistic manner. Thus, our study has provided a new rationale for further exploration in this area. As measurements of Cre and Cysc were clinically inexpensive and accessible, the CCR may be a prospective and time-efficient marker for identification of cardiovascular risk in individuals.

Cre, a metabolic by-product of muscle tissue, is typically eliminated by the kidneys through urine. Given its status as a by-product of muscle metabolism, the Cre concentrate is closely correlated with muscle mass. Cysc is a protein produced by nucleated cells and is primarily used as a marker of glomerular filtration rate (GFR), which is minimally affected by mass metabolism (31–35). Given the greater reliance of Cre levels on muscle mass than Cysc levels, the computation of the ratio of Cre to Cysc, which allows for a degree of correction for errors in a single marker due to aberrant renal function or changes in muscle mass, represents a more stable and reliable method for the evaluation of muscle mass (36).

In addition to reflecting bone and muscle mass, CCR may also serve as an indicator of an inflammatory state. In instances of chronic infection, the formation of Cre may be suppressed. Consequently, reduced levels of Cre are frequently associated with an inflammatory response (15). An elevated Cysc level is associated with an exacerbation of the inflammatory state. In addition to its role as a sensitive marker of renal function, Cysc is also a reliable indicator of the body’s overall inflammatory burden (37). Furthermore, CCR is indicative of chronic inflammation, with elevated CCR values correlating with heightened inflammatory negativity (38). Furthermore, inflammation has been demonstrated to be a pivotal factor in the development and progression of CVD, particularly in conditions such as atherosclerosis, coronary artery disease, and heart failure (39–42). Thus, fluctuations in CCR levels, particularly elevated Cysc, may indicate that the organization is in a state of chronic inflammation and that this may be associated with the onset and progression of CVDs.

While the present study focused on the cardiovascular predictive value of CCR, the potential impact of chronic kidney disease (CKD) as an important confounder is acknowledged. CKD has been identified as a risk factor for CVD (43), occupying a significant position within the complex network of pathophysiological processes associated with inflammation and CVD (44). Renal hypoplasia has been demonstrated to result in the accumulation of uremic retention molecules within the body (45), thereby inducing oxidative stress. Oxidative stress, in turn, has been shown to promote inflammatory cell recruitment through the activation of the NF-κB pathway (46). Concurrently, renal hypoplasia has been observed to induce vascular calcification and thickening of the aortic wall (47). These phenomena have been demonstrated to result in endothelial cell dysfunction and vascular smooth muscle cell dysfunction, thereby accelerating the pathogenesis of cardiovascular disease (45). This CKD-inflammation-CVD triad may partially explain the association between CCR and CVD in this study: Cystatin C, a GFR marker, exhibits elevated levels that directly reflect renal impairment (48); CKD-related muscle atrophy affects creatinine metabolism, thereby interfering with the interpretation of CCR (49). However, after adjusting for CRE-based eGFR, CCR maintained an independent association with CVD risk (OR=0.78, 95%CI:0.68-0.90), a robustness that suggests that the predictive value of CCR is not exclusively mediated by renal function and that muscle metabolism may influence CVD risk through pathways other than renal function (e.g., anti-inflammatory effects). This finding has the potential to generate novel concepts for the development of personalized cardiovascular risk assessment strategies for diverse renal function states.

The present study has several notable strengths. First and foremost, our findings indicated that CCR may offer a novel approach to the surveillance of CVD, particularly in the elderly population. To the best of our knowledge, this was the inaugural study to examine the correlation between CCR and CVD. Subsequently, we adjusted for a multitude of potential confounding variables to ensure robust results. Thirdly, the integration of cross-sectional data to establish correlations at a specific point in time and the incorporation of longitudinal data to elucidate the causal relationships served to reinforce the compelling nature of the findings. Nevertheless, it was imperative to recognize that our study was not without its inherent limitations. (i)Population limitations: As the study population was limited to Chinese older adults, the findings may not be applicable to other populations. (ii)Self-reporting bias: CVD diagnosis relied on self-reporting, and the absence of medical records may have affected the accuracy of the results. (iii) Selection bias: Subjects with missing Cre and Cysc data and incomplete CVD data were eliminated, which may cause bias in selection and restrict the application of the findings. It would be prudent to consider that such limitations may influence the prevalence and precision of the research results, and that additional research may be required to confirm them.

## 5 Conclusion

The results demonstrated that a relatively high CCR was associated with a reduced risk of CVD in middle-aged and elderly Chinese individuals. Given the extensive clinical utilization of Cre and Cysc, the CCR, as a cost-effective marker, may prove effective in identifying individuals at risk of CVD, thereby facilitating early intervention to slow the progression of CVD. However, further verification of these results and assessment of their suitability for use in a wider population are necessary.

## Data Availability

The original contributions presented in the study are included in the article/**Supplementary Material**. Further inquiries can be directed to the corresponding author.
